# Text Messaging Support for Patients Diagnosed With Impaired Glucose Tolerance During Pregnancy: Nonrandomized Pre-Post Implementation Study Assessing Impact on Postpartum Transitions of Care

**DOI:** 10.2196/76493

**Published:** 2026-06-09

**Authors:** Natasha R Kumar, Whitney Bender, Celeste Durnwald

**Affiliations:** 1 Department of Obstetrics and Gynecology, Michigan Medicine, University of Michigan, 1500 E Medical Center Drive, Ann Arbor, MI, 48109, United States, 1 717-712-8582; 2 Department of Obstetrics and Gynecology, Sidney Kimmel Medical College, Thomas Jefferson University Hospital, Philadelphia, PA, United States; 3 Department of Obstetrics and Gynecology, Perelman School of Medicine, University of Pennsylvania Health System, Philadelphia, PA, United States

**Keywords:** postpartum transitions of care, impaired glucose tolerance, obstetrics, quality improvement, mobile health interventions

## Abstract

**Background:**

Patients with impaired glucose tolerance (IGT) identified during pregnancy who do not develop gestational diabetes mellitus (GDM) often do not receive additional interventions for their long-term metabolic risks.

**Objective:**

This nonrandomized pre-post implementation study reports the design process and initial program evaluation for Better Follow-up of Impaired Glucose Tolerance (BRIDGE), a 12-week text-based postpartum support program promoting hemoglobin A_1c_ (HbA_1c_) completion and primary care provider (PCP) visit scheduling for patients diagnosed with IGT during pregnancy, assessing improvement in desired postpartum transition milestones. The 19-month program was divided into 2 arms lasting 9.5 months each, BRIDGE− (SMS text messaging support alone; October 2021-July 2022) and BRIDGE+ (SMS text messaging and IGT-focused postpartum visit; July 2022-April 2023). We aimed to assess whether BRIDGE improved desired postpartum transition milestones.

**Methods:**

Patients were eligible for BRIDGE if they received prenatal care at the study site (a northeastern US academic tertiary care center), were diagnosed with IGT during pregnancy, never developed GDM, and could receive English text messages. We performed a program evaluation using a pre/postimplementation design, comparing outcomes for the BRIDGE population to a 19-month historical population. Primary outcomes were (1) completion of HbA_1c_ testing by 1 year postpartum and (2) PCP visit scheduling by 12 weeks postpartum. A comparative analysis between BRIDGE− and BRIDGE+ was performed. Multivariable logistic regressions controlled for the history of IGT after stepwise backward elimination.

**Results:**

In the program evaluation, 503 individuals were included (n=342 in historical population, n=82 in BRIDGE− population, and n=79 in BRIDGE+ population), with similar demographic and clinical characteristics across populations. A total of 212 individuals were screened for eligibility in BRIDGE, and 161 individuals participated in the program. BRIDGE participants had increased odds of HbA_1c_ completion by 1 year postpartum (39.8% vs 12.5%; adjusted odds ratio [aOR] 4.28, 95% CI 2.71‐6.78) and PCP visit scheduling (31.0% vs 12.0%; aOR 9.58, 95% CI 4.39‐20.9) compared to the historical population. BRIDGE+ patients were more likely to complete HbA_1c_ testing by 12 weeks postpartum than BRIDGE− participants. Most patients attended scheduled PCP visits, but rates of IGT counseling at PCP visits were low.

**Conclusions:**

Individuals with IGT rarely receive targeted interventions during pregnancy or delivery hospitalization. This innovative study demonstrates that individuals with IGT have high rates of uptake for postpartum SMS text messaging support, which tripled completion rates of HbA_1c_ screening within 1 year postpartum and doubled the scheduling rate for PCP visits by 12 weeks postpartum. While attendance at scheduled PCP visits was very high, <60% of PCP visits included IGT counseling, highlighting key improvement areas in the quality of postpartum transitions to primary care. While a randomized trial is needed to ascertain definitive impact, SMS text messaging support may be an effective tool to improve postpartum transitions of care for this underserved population.

## Introduction

### Background

According to the 2020 National Diabetes Statistics Report, approximately 1 in 3 American adults has impaired glucose tolerance (IGT) or prediabetes, defined as hemoglobin A_1c_ (HbA_1c_) of 5.7 to 6.4 [1]. IGT is often considered an intermediate state of abnormal glucose metabolism that exists between normal glucose homeostasis and diabetes. The natural history of IGT is variable and may include normalization, persistence of impaired tolerance, or progression to diabetes [2-4]. Up to 70% of patients with IGT may ultimately develop diabetes without intervention [5]. Given the variable natural history and the ability to alter disease trajectory with behavioral modifications, individuals with IGT are ideal candidates for diabetes prevention efforts [6,7]. Pregnancy is a time when individuals enter the health care system and may be motivated to focus on their health. As a result, obstetricians can play a role in promoting overall health, impacting both future pregnancy and long-term health outcomes.

As the prevalence of preexisting and gestational diabetes mellitus (GDM) among pregnant people has increased in recent years [8,9], universal HbA_1c_ screening in early pregnancy has been proposed to identify undiagnosed pre-existing diabetes as well as to screen for early-onset GDM [10,11]. In a retrospective cohort at a single institution, 12% of patients had IGT in early pregnancy but never developed GDM [12]. Patients with IGT in pregnancy are at risk of both adverse pregnancy outcomes and long-term health outcomes, even if they never develop GDM. Although there are relatively few studies of pregnant patients with IGT and without GDM, several studies have demonstrated an increased risk of spontaneous preterm birth less than 37 weeks [13]. A singular cohort study has also shown a potentially increased risk of hypertensive disorders of pregnancy (HDP), major congenital anomalies, shoulder dystocia, large for gestational age births, and perinatal death in this population [13]. The American Diabetes Association recommends that patients with IGT complete HbA_1c_ screening for the development of diabetes every 1 to 2 years [14]. Pregnant patients diagnosed with GDM receive counseling on dietary and physical activity recommendations, antenatal glucose monitoring, counseling on long-term metabolic risks, and postpartum glucose testing. In contrast, there are no established guidelines for the prenatal or postpartum management of pregnant patients with IGT and without GDM. They may not receive dietary and exercise counseling, counseling on long-term metabolic risks, postpartum glucose testing, or a formal transition of care to a primary care physician or endocrinologist. This can create gaps in care with implications for future pregnancy and future health outcomes. In previously unpublished data from our institution, an exploratory analysis of long-term outcomes for 100 patients with IGT in pregnancy at a single institution demonstrated that 71% of patients did not have a visit with a primary care provider (PCP) in 1 to 3 years after delivery. Of the 29 patients with PCP visits, nearly half (48.3%) did not obtain repeat HbA_1c_ values to determine if there was persistence of IGT. In half of the cases, this was due to a lack of ordering by the PCP. Of the 15 patients who completed repeat HbA_1c_ testing, 93.3% remained more than 5.7% 1 to 3 years after delivery. There were also 14 repeat pregnancies in this cohort, 78.6% of which continued to demonstrate IGT during early pregnancy screening.

SMS text messaging support programs have been used effectively during the postpartum period to support transitions of care. A randomized controlled trial of an SMS text messaging support program prompting primary care transitions demonstrated significant improvement in primary care visit completion, as well as fewer postpartum readmissions and increased receipt of services, such as blood pressure assessments and screenings for mood symptoms [15]. Other SMS text messaging support programs have focused on the completion of recommended clinical assessments postpartum for patients with pregnancy complications, such as hypertensive disorders of pregnancy or gestational diabetes, with many programs reporting improved engagement with SMS text messaging support [16-20].

Based on our institutional data demonstrating clinical need, our team developed a program called Better Follow-up of Impaired Glucose Tolerance (BRIDGE) to address postpartum transitions of care for patients diagnosed with IGT during pregnancy. Here, we present a single institution nonrandomized pre-post implementation study, including (1) the design process for BRIDGE and a program evaluation, (2) a comparison of clinical outcomes for BRIDGE patients to a historical population, and (3) a comparative analysis examining differences in outcomes between 2 programmatic strategies used within BRIDGE. We hypothesize that the addition of SMS text messaging support and an IGT-focused postpartum visit will improve the completion of desired postpartum milestones for this population (ie, HbA_1c_ completion, primary care visit scheduling). Based on the findings noted in this study, we anticipate performing a future randomized trial to definitively assess the impact of this intervention.

### Intervention

#### Overview of Program Design

Our team developed a program called BRIDGE to address postpartum transitions of care for patients diagnosed with IGT during pregnancy. The first half of BRIDGE (BRIDGE−; October 2021-July 2022) enrolled participants in biweekly text messages between 0 and 12 weeks postpartum, including educational content and reminders to complete desired postpartum transition milestones, namely (1) completing HbA_1c_ assessment and (2) scheduling a visit with a PCP. During the last half of the program (BRIDGE+; July 2022-April 2023), participants were offered an additional postpartum visit for standardized IGT education in addition to the SMS text messaging support.

#### Text Message Design and Content

**Figure 1.**
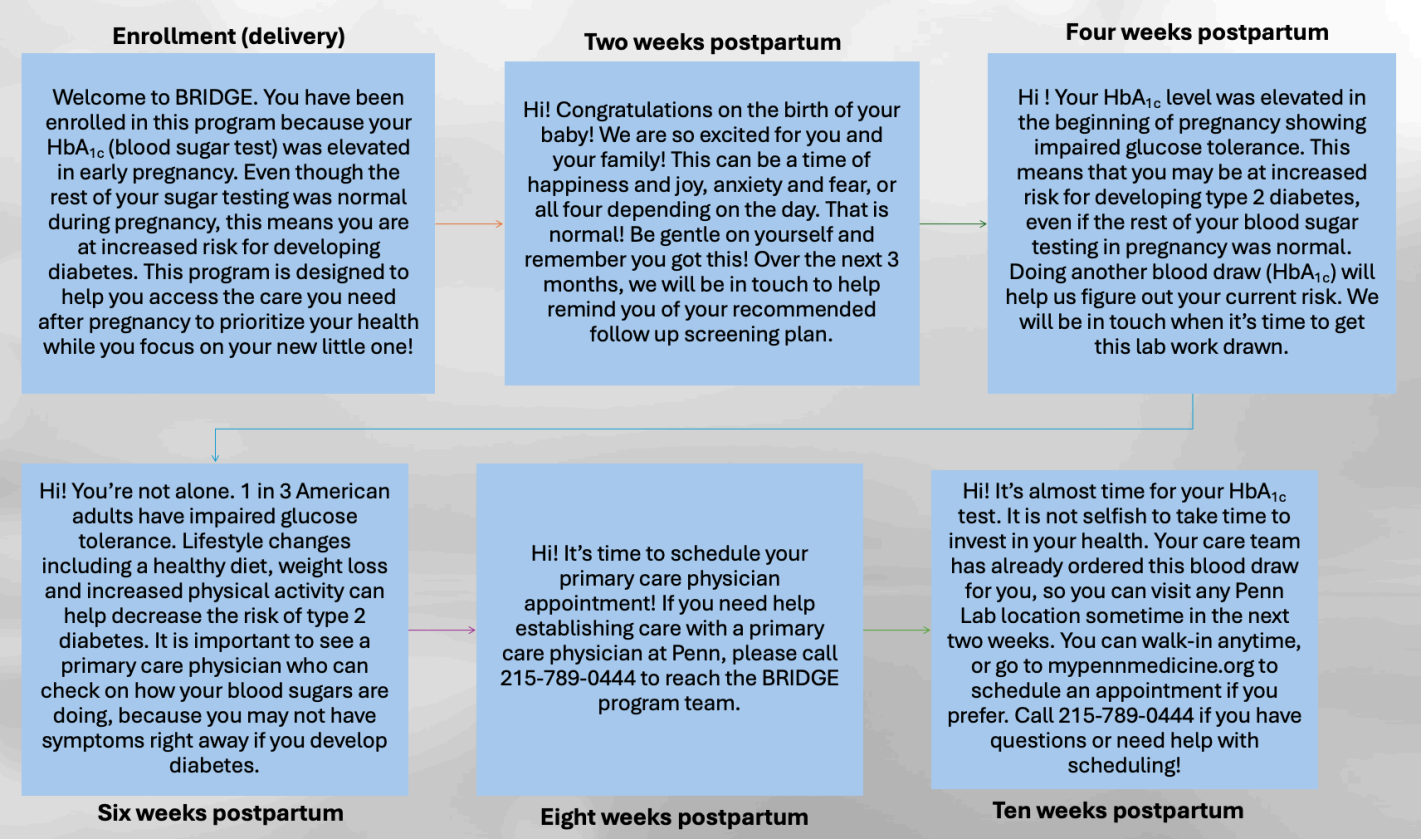
Overview of Better Follow-up of Impaired Glucose Tolerance (BRIDGE) text message content and timing. HbA_1c_: hemoglobin A_1c_.

The BRIDGE SMS text messaging program was designed in collaboration with multiple expert groups within our health care institution, as well as lived-experience experts. First, we partnered with Way2Health (W2H), a web-based platform providing technology infrastructure for sustainable behavior change interventions to build the SMS text messaging support program [21-23]. W2H infrastructure is integrated into the electronic medical record (EMR) system at our institution, which allows providers to confirm enrollment in BRIDGE and review text message content if desired. To optimize the frequency and content of messaging in the program, we collaborated with the Nudge Unit, a behavioral design team embedded within our institution’s health care system (Figure 1). Finally, we reviewed the planned messages with a lived experience expert to confirm that the language and content of the messaging were appropriate and affirming.

#### Primary Care Physician Scheduling and Follow-Up

Our study team held preimplementation planning meetings with both our prenatal care provider teams and the primary care teams to optimize workflows for patient enrollment and engagement with the program.

To facilitate participants’ success with expected milestones, we developed an expedited pathway for PCP referral through the BRIDGE program within our health care system. We used a referral order with a specific indication related to BRIDGE within our EMR. These BRIDGE referrals to primary care were reviewed by the administrative team for the primary care offices on a bimonthly or monthly basis to expedite scheduling.

Monthly implementation meetings were held to review programmatic outcomes, and certain implementation strategies were added or removed to address specific barriers communicated by our prenatal care providers or PCPs, as well as patients. For example, referrals were integrated into a virtual social work support program offered by the institution to address patients’ reports regarding social drivers of health that were limiting their engagement with care.

Importantly, the ongoing implementation meetings led to a significant strategic shift at the midway point of the program where an IGT-focused postpartum visit (iPPV) was added, during which prenatal providers discussed the long-term metabolic risks associated with IGT and reiterated the importance of HbA_1c_ completion and PCP visit scheduling. The addition of the iPPV was based on feedback from both patients, who desired to continue care in settings where they had established trust during pregnancy, as well as the primary care team, which reported workforce capacity and insurance challenges. This marked the transition from BRIDGE− to BRIDGE+.

## Methods

### Pre-Post Implementation Study Design

This is a pre-post implementation study of BRIDGE, an SMS text messaging support program promoting postpartum transitions of care for patients with IGT during pregnancy. A program evaluation was performed assessing differences in outcomes between the BRIDGE population and a historical population, as well as between 2 groups enrolled in 2 different programmatic strategies of BRIDGE, as described previously in the *Overview of Program Design* section.

### Participants

Patients were eligible for the program if they had an HbA_1c_ value of 5.7 to 6.4 prior to 20 weeks of gestation, completed third-trimester glucose testing, did not receive a diagnosis of GDM, had a singleton pregnancy, delivered at our hospital, and were English-speaking. BRIDGE was implemented at a single tertiary care institution, with approximately 4000 deliveries per year, in the northeastern United States. Opt-out enrollment of eligible patients was performed manually by the study team at the time of their delivery. Patients received a text message with a description of the program, recommended milestones, and the option to decline participation.

For our historical population, we selected all patients who met identical eligibility criteria to BRIDGE participants and delivered between June 2018 and December 2019. We selected a continuous 19-month time period (identical to the BRIDGE program duration) that preceded the COVID-19 pandemic in March 2020 (given the disruptions to typical care during that time).

### Outcomes

The 2 primary outcomes for the BRIDGE program were (1) completion of an HbA_1c_ test and (2) scheduling of a visit with a PCP by 12 weeks postpartum. Given the paucity of national recommendations regarding follow-up for IGT identified in early pregnancy, these postpartum transition milestones were determined based on expert opinions from our maternal fetal medicine, primary care, and endocrinology teams. When completing the comparative analysis with the historical population, HbA_1c_ completion was assessed by 1 year postpartum rather than by 12 weeks postpartum to provide an appropriate comparison with the standard of care during the historical period. For our analysis comparing the 2 programmatic strategies in BRIDGE− and BRIDGE+, we assessed HbA_1c_ completion by 12 weeks postpartum, which is the programmatic goal, as well as by 1 year postpartum, which is aligned with national recommendations from some organizations such as the American Diabetes Association [24]. To account for potential bias caused by missing data, because the authors were unable to use retrospective chart abstraction to identify data outside of the health care system, reporting of these outcomes was stratified among patients with an identified PCP within the network versus patients with an identified PCP outside of the network. In addition, the evaluation was adjusted for demographic and clinical characteristics that differed between the study groups.

Secondary outcomes included other process outcomes, such as PCP visit attendance and the inclusion of IGT counseling during PCP visits (for in-network patients where these data were available in our EMR). We also reported descriptive data regarding iPPV scheduling and attendance for BRIDGE+ participants only. We stratified all outcomes related to PCP visit scheduling and attendance by patients who had in-network PCPs versus all patients due to the insurance and workforce capacity challenges faced by patients who did not have established PCPs within our health care system, as well as considerations regarding potential missing data.

The data were collected by the study team on participant demographics and clinical characteristics, as well as primary and secondary outcomes, through an EMR review.

### Sample Size

With a sample size of 246 patients, we would be powered to detect an improvement in PCP visit scheduling from an assumed baseline of 30% based on prior chart review at our institution as well as existing literature [25] to 45% with 80% power and an α level of .05. With an annual birth volume of 4000 patients per year and baseline data at the institution demonstrating that 12% of the population has IGT in early pregnancy, the study anticipated the need to collect data for at least 7 months to accrue a sufficient sample size. All analyses were performed using Stata version 17.0 (StataCorp LLC).

For this nonrandomized study, the CONSORT (Consolidated Standards of Reporting Trials) extension for pilot and feasibility trials was used to report the findings including the abstract extension [26,27].

### Statistical Methods

Race and ethnicity were self-reported. For the primary analysis comparing the BRIDGE population to a historical population, bivariate analyses comparing clinical and demographic characteristics between our BRIDGE− or BRIDGE+ and historical populations were performed using 2-tailed *t* tests for continuous variables and chi-squared tests for categorical variables. We also assessed 95% CIs for point estimates of these characteristics in each group using the Wilson score intervals. Multivariable logistic regressions comparing the odds of all assessed outcomes in the BRIDGE versus historical population included all variables with *P*<.20 in the bivariate analyses. Backward stepwise regression was used to create parsimonious models using *P*>.20 for elimination. An identical strategy was used to develop models for comparative analysis assessing differences in outcomes between our BRIDGE− and BRIDGE+ populations. For both analyses, models comparing outcomes across populations ultimately only included a history of IGT in prior pregnancy as a covariate. Multiple imputation analysis was used to address missing data at random for primary care visit scheduling and attendance.

### Ethical Considerations

The project was designated as a quality improvement project and deemed exempt from review by the University of Pennsylvania Institutional Review Board, as (1) this intervention did not guide any clinical decisions regarding care and was intended solely to improve care delivery within the health care system, and (2) participants were not randomized to the intervention and were intended to obtain direct benefits from the intervention. Informed consent was not required for this quality improvement initiative. Deidentified data were collected for retrospective review and stored on secure institutional servers. Participants were not compensated for their participation in this initiative. There is no identification of any individual participants in any manuscript images or supplementary materials.

## Results

### Participant Recruitment

**Figure 2.**
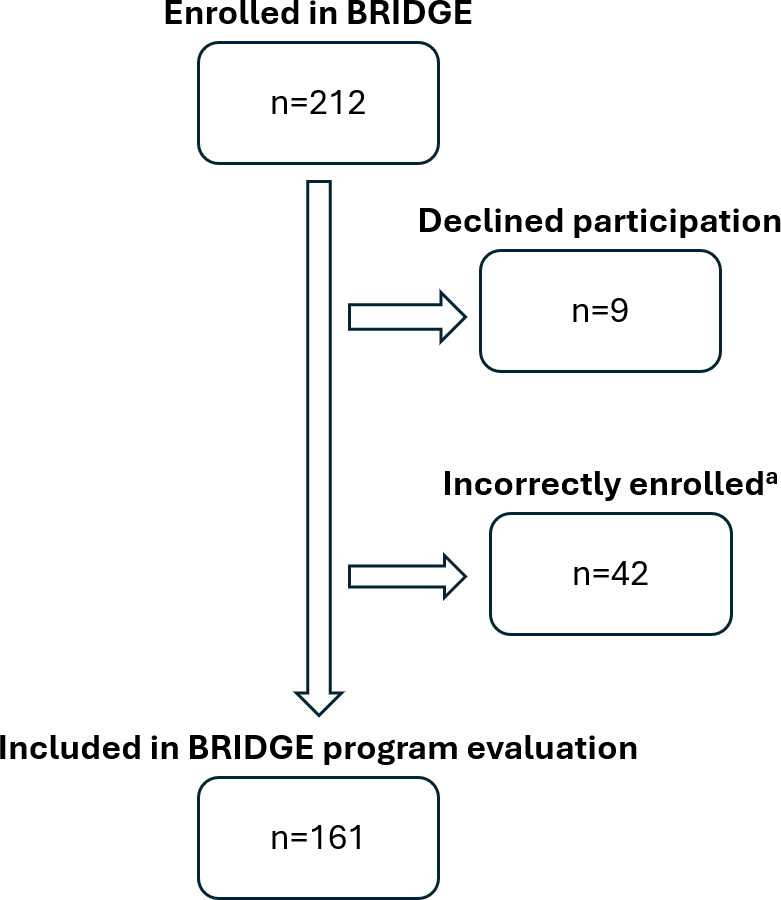
Participant flow diagram for Better Follow-up of Impaired Glucose Tolerance (BRIDGE) enrollment and program evaluation. ^a^Participants who were incorrectly enrolled did not meet eligibility requirements, that is, individuals for whom English was not a primary language, individuals diagnosed with gestational diabetes, or did not meet criteria for impaired glucose tolerance (IGT) in early pregnancy, and individuals who did not deliver at our hospital.

The recruitment of participants occurred between October 2021 and April 2023 and was stopped due to funding constraints, although the target sample size was not achieved. A convenience sampling of patients meeting the eligibility criteria for the intervention and presenting to prenatal clinics within the health care system over the study time period was performed. Providers had the option of directly enrolling patients from the clinic. In addition, on a weekly basis, the study team received an automated report from the health care system analytics team with all newly eligible participants. After reviewing the medical records to confirm eligibility, the study team manually enrolled all eligible patients. The team also reviewed the EMR of any participants added directly by clinicians and removed participants deemed ineligible. A participant flow diagram was included to demonstrate the number of individuals enrolled and ultimately included in the program evaluation (Figure 2).

### Demographic and Clinical Characteristics of the Study Populations

In total, 161 participants were enrolled in the BRIDGE program, compared to 342 participants in the historical population (Table 1). A higher proportion of the BRIDGE population had a personal history of IGT and polycystic ovarian syndrome compared to the historical population (Table 1). There were 82 participants in the BRIDGE− arm and 79 participants in the BRIDGE+ arm (Table 2). The BRIDGE− and BRIDGE+ populations had similar demographic and clinical characteristics.

**Table 1.** Demographic and clinical characteristics of patients with impaired glucose tolerance (IGT) in a historical population (June 2018-December 2019) versus those enrolled in SMS text messaging support program (BRIDGE^a^; October 2021-April 2023).

Characteristics	Historical population (n=342)	BRIDGE^b^ (N=161)
Demographics		
Age (y), median (IQR^b^)	31 (27-35)	32 (28-36)
Insurance, n (%; 95% CI)		
Private	159 (46.5%; 41.3%-51.8%)	65 (40.4%; 33.1%-48.1%)
Public	179 (52.3%; 47%-57.6%)	96 (59.6%; 51.9%-66.9%)
None	4 (1.2%; 0.5%-3%)	0 (0%; 0%-2.3%)
Race, n (%; 95% CI)		
White	36 (10.5%; 7.7%-14.2%)	11 (6.8%; 3.9%-11.8%)
Black	256 (74.9%; 70%-79.2%)	131 (81.4%; 74.6%-86.6%)
Asian	20 (5.8%; 3.8%-8.9%)	10 (6.2%; 3.4%-11.1%)
Other or unknown	30 (8.8%; 6.2%-12.2%)	9 (5.6%; 2.6%-10.4%)
Hispanic ethnicity, n (%; 95% CI)	16 (4.7%; 2.9%-7.5%)	7 (4.3%; 1.8%-8.8%)
Scant prenatal care (<5 visits), n (%; 95% CI)	6 (1.8%; 0.8%-3.8%)	2 (1.3%; 0.3%-4.4%)
Late prenatal care initiation (>16 weeks), n (%; 95% CI)	9 (2.6%; 1.4%-4.9%)	8 (5.0%; 2.6%-9.6%)
Nulliparous, n (%; 95% CI)	107 (31.3%; 26.6%-36.4%)	44 (27.3%; 21.0%-34.7%)
Primary care provider, n (%; 95% CI)		
In network	118 (34.5%; 30.0%-40.0%)	54 (33.5%; 26.7%-41.1%)
Out of network	118 (34.5%; 30.0%-40.0%)	46 (28.6%; 22.2%-36.0%)
None	106 (31.0%; 26.3%-36.1%)	61 (37.9%; 30.8%-45.6%)
Clinical characteristics, n (%; 95% CI)		
History of prior IGT^h^	35 (10.2%; 7.45%-13.9%)	40 (24.8%; 18.8%-32.1%)
History of GDM^d^ in prior pregnancy	5 (1.5%; 0.6%-3.4%)	2 (1.3%; 0.3%-4.4%)
History of macrosomia in prior pregnancy	3 (0.8%; 0.2%-2.5%)	4 (2.5%; 0.9%-6.2%)
History of IUFD^e^ >20 weeks gestation	1 (0.3%; 0.05%-1.6%)	0 (0%; 0%-2.3%)
Family history of diabetes	83 (24.3%; 20.0%-29.1%)	49 (30.4%; 23.9%-37.9%)
History of PCOS^f^	4 (1.2%; 0.4%-3.0%)	11 (6.8%; 3.9%-11.8%)
History of metformin use	7 (2.0%; 1.0%-4.2%)	7 (4.3%; 2.1%-8.7%)

a BRIDGE: Better Follow-up of Impaired Glucose Tolerance.

b Missing values: scant prenatal care (n=1 in BRIDGE population); late prenatal care (n=1 in BRIDGE population); GDM in prior pregnancy (n=1 in BRIDGE population).

c IGT: impaired glucose tolerance.

d GDM: gestational diabetes mellitus.

e IUFD: intrauterine fetal death.

f PCOS: polycystic ovarian syndrome.

**Table 2.** Demographic and clinical characteristics of patients with impaired glucose tolerance (IGT) enrolled in SMS text messaging support (BRIDGE−^a^; October 2021-July 2022) versus SMS text messaging support + IGT-focused postpartum visit (BRIDGE+; July 2022-April 2023).

Characteristics	BRIDGE–^b^ (n=82)	BRIDGE+ (n=79)
Demographics		
Age (y), median (IQR^b^)	32 (28-36)	33 (29-36)
Insurance, n (%; 95% CI)		
Private	32 (39.0%; 29.2%-49.8%)	33 (41.8%; 31.5%-52.8%)
Public	50 (61.0%; 50.2%-70.8%)	46 (58.2%; 47.2%-68.4%)
Race, n (%; 95% CI)		
White	3 (3.7%; 1.3%-10.2%)	8 (10.1%; 5.2%-18.7%)
Black	72 (87.8%; 79%-93.2%)	59 (74.7%; 64.1%-83%)
Asian	4 (4.9%; 1.9%-11.9%)	6 (7.6%; 3.5%-15.6%)
Other or unknown	3 (3.7%; 1.3%-10.2%)	6 (7.6%; 3.5%-15.6%)
Hispanic ethnicity, n (%; 95% CI)	2 (2.4%; 0.7%-8.5%)	5 (6.3%; 2.7%-14%)
Scant prenatal care (<5 visits), n (%; 95% CI)	0 (0%; 0%-4.5%)	2 (2.5%; 0.7%-8.9%)
Late prenatal care initiation (>16 weeks), n (%; 95% CI)	4 (4.9%; 1.9%-12%)	4 (5.1%; 2.0%-12.5%)
Nulliparous	21 (25.6%; 17.4%-36%)	23 (29.1%; 20.3%-39.9%)
Primary care provider		
In network	24 (29.3%; 20.5%-39.9%)	30 (38.0%; 28.1%-49%)
Out of network	22 (26.8%; 18.4%-37.3%)	24 (30.4%; 21.3%-41.2%)
None	36 (43.9%; 33.7%-54.7%)	25 (31.6%; 22.4%-42.5%)
Clinical characteristics, n (%; 95% CI)		
History of prior IGT	16 (19.5%; 12.4%-29.4%)	24 (30.3%; 21.3%-41.2%)
History of GDM^c^ in prior pregnancy	0 (0%; 0%-4.5%)	2 (2.5%; 0.7%-8.9%)
History of macrosomia in prior pregnancy	3 (3.7%; 1.3%-10.2%)	1 (1.3%; 0.2%-6.8%)
History of IUFD^d^ >20 weeks gestation	0 (0%; 0%-4.5%)	0 (0%; 0%-4.6%)
Family history of diabetes	23 (28.0%; 19.5%-38.6%)	26 (32.9%; 23.6%-43.9%)
History of PCOS^e^	5 (6.1%; 2.6%-13.5%)	6 (7.6%; 3.5%-15.6%)
History of metformin use	5 (6.1%; 2.6%-13.5%)	2 (2.5%; 0.7%-8.9%)

a BRIDGE: Better Follow-up of Impaired Glucose Tolerance.

b Missing values: scant prenatal care (n=1 in BRIDGE− population); late prenatal care (n=1 in BRIDGE− population); GDM in prior pregnancy (n=1 in BRIDGE− population).

c GDM: gestational diabetes.

d IUFD: intrauterine fetal death.

e PCOS: polycystic ovarian syndrome.

### Program Evaluation of BRIDGE Using Historical Population for Comparison

BRIDGE participants had increased odds of HbA_1c_ completion by 1 year postpartum (39.8% vs 12.5%; adjusted odds ratio [aOR] 4.28, 95% CI 2.71‐6.78) and PCP visit scheduling (31.0% vs 12.0%; aOR 9.58, 95% CI 4.39‐20.9) compared to the historical population (Table 3). The difference in PCP visit scheduling remained statistically significant in the multiple imputation analysis (aOR 9.49, 95% CI 4.60‐19.6; Multimedia Appendix 1). This was also true among the subgroup of patients with in-network PCPs (27.3% vs 12.0%; aOR 8.30, 95% CI 3.77‐18.2). Importantly, in the historical population, every patient who scheduled a PCP visit had an established in-network PCP during pregnancy; in contrast, in the BRIDGE population, 6 out of the 50 (12%) patients who scheduled a PCP visit did not already have an established in-network PCP.

**Table 3.** Comparing postpartum transitions of care for patients with impaired glucose tolerance (IGT) in a historical population (June 2018-December 2019) versus SMS text messaging support Better Follow-up of Impaired Glucose Tolerance (BRIDGE) population (October 2021-April 2023).

Postpartum transitions	Historical population (n=342)	BRIDGE population (N=161)	aOR^a^ (95% CI)
HbA_1c_^b^ completion by 1 year postpartum, n (%; 95% CI)	43 (12.5%; 9.5%-16.5%)	64 (39.8%; 32.5%-47.5%)	4.28 (2.71-6.78)
PCP^c^ visit scheduling, n (%; 95% CI)			
In network PCPs^d^	41 (12.0%; 9%-15.9%)	44 (27.3%; 21%-34.7%)	8.30 (3.77-18.2)
All patients^e^, n (%; 95% CI)	41 (12.0%; 9%-15.9%)	50 (31.0%; 24.4%-38.6%)	9.58 (4.39-20.9)
PCP visit attendance, n (%; 95% CI^f^)			
In network PCPs	38 (92.7%; 80.6%-97.5%)	42 (95.4%; 84.9%-98.7%)	1.81 (0.28-11.7)
All patients	38 (92.7%; 80.6%-97.5%)	48 (96%; 86.5%-98.9%)	2.11 (0.33-13.6)
PCP visits with IGT counseling, n (%; 95% CI)			
In network PCPs	15 (39.5%; 25.6%-55.3%)	21 (50.0%; 35.5%-64.5%)	1.45 (0.59-3.56)
All PCPs	15 (39.5%; 25.6%-55.3%)	26 (54.2%; 40.3%-67.4%)	1.65 (0.68-3.98)

a aOR: adjusted odds ratios for history of prior IGT.

b HbA_1c_: hemoglobin A_1c_.

c PCP: primary care physician.

d A limited number of patients had in-network PCPs (n=119 for patients in historical population; n=54 for patients in BRIDGE).

e The data on PCP visit scheduling and attendance for patients with out-of-network PCPs were obtained via electronic medical record review (of postpartum notes) for both historical and BRIDGE populations. Phone interviews were also attempted for BRIDGE patients to obtain self-reported data, which were limited by a low response rate (n=10 interviews total).

f There were missing data for PCP visit scheduling and attendance (n=221 in historical population; n=101 in BRIDGE population).

There were remarkably high levels of attendance of PCP visits among those who scheduled PCP visits, across both the historical and BRIDGE populations (95% CI 92.7%‐96% of scheduled visits). However, rates of IGT counseling during PCP visits were relatively low in both the historical and BRIDGE populations (95% CI 39.5%‐54.2% of visits).

### Comparative Analysis Between BRIDGE− and BRIDGE+ Arms

In comparing the BRIDGE− to BRIDGE+ arms of the program, the BRIDGE+ population had slightly improved odds of HbA_1c_ completion by 12 weeks postpartum (36.7% vs 19.5%; aOR 2.31, 95% CI 1.12‐4.74), though this positive effect using the BRIDGE+ strategy did not persist by 1 year postpartum (48.1% vs 31.7%; aOR 1.88, 95% CI 0.98‐3.61; Table 4). There was no difference in PCP visit scheduling between the BRIDGE− and BRIDGE+ program strategies (32.9% vs 29.3%; aOR 0.46, 95% CI 0.11‐2.00), which remained consistent in multiple imputation analysis (aOR 0.49, 95% CI 0.10‐2.25; Multimedia Appendix 1). This was similar when looking at PCP scheduling among patients with established in-network PCPs specifically (29.1% vs 25.6%; aOR 0.47, 95% CI 0.11‐2.09). A similar proportion of patients who did not have established in-network PCPs were able to schedule a PCP visit in both the BRIDGE− and BRIDGE+ populations.

**Table 4.** Comparing the effectiveness of SMS text messaging support (BRIDGE−^a^) versus SMS text messaging support + impaired glucose tolerance (IGT)-focused postpartum visit (BRIDGE+) in completing postpartum transitions of care for patients with IGT.

Postpartum transitions	BRIDGE− (n=82)	BRIDGE+ (n=79)	aOR^b^ (95% CI)
HbA_1c_^c^ completion at 12 weeks postpartum, n (%; 95% CI)	16 (19.5%; 12.4%-29.4%)	29 (36.7%; 26.9%-47.7%)	2.31 (1.12-4.74)
HbA_1c_ completion at 1 year postpartum, n (%; 95% CI)	26 (31.7%; 22.6%-42.4%)	38 (48.1%; 37.4%-58.9%)	1.88 (0.98-3.61)
PCP^d^ visit scheduling^e^, n (%; 95% CI)			
In network PCPs^e^	21 (25.6%; 17.4%-36%)	23 (29.1%; 20.3%-39.9%)	0.47 (0.11-2.09)
All patients^f^	24 (29.3%; 20.5%-39.9%)	26 (32.9%; 23.6%-43.9%)	0.46 (0.11-2.00)
PCP visit attendance^e^, n (%; 95% CI)			
In network PCPs	19 (90.4%; 71.1%-97.3%)	23 (100%; 85%-100%)	—^g^
All patients	22 (91.6%; 74.2%-97.7%)	26 (100%; 87%-100%)	—
PCP visits with IGT counseling, n (%; 95% CI)			
In network PCPs	10 (52.6%; 31.7%-72.7%)	11 (47.8%; 29.2%-67%)	0.70 (0.23-2.73)
All PCPs	12 (54.5%; 34.7%-73.1%)	14 (53.8%; 40.8%-77.8%)	0.98 (0.30-3.20)
IGT-focused postpartum visit^h^, n (%; 95% CI)			
Appointment scheduled	—	47 (59.5%; 48.5%-69.6%)	—
Appointment attended	—	23 (48.9%; 35.3%-62.8%)	—

a BRIDGE: Better Follow-Up of Impaired Glucose Tolerance.

b aOR: adjusted odds ratios for history of prior IGT.

c HbA_1c_: hemoglobin A_1c._.

d PCP: primary care physician.

e There were missing data for PCP visit scheduling and attendance (n=55 in BRIDGE− population, n=46 in BRIDGE+ population).

f A limited number of patients had in-network PCPs (n=119 for patients in historical population; n=24 for patients in BRIDGE−; n=30 for patients in BRIDGE+).

g Not applicable.

h The data on PCP visit scheduling and attendance for patients with out-of-network PCPs were obtained via electronic medical record review (of postpartum notes) for both historical and BRIDGE populations. Phone interviews were also attempted for BRIDGE patients to obtain self-reported data, which were limited by low response rate (n=10 interviews total).

i Only BRIDGE+ participants were eligible for this visit (n=79).

Once again, there were remarkably high attendance rates for scheduled PCP visits (91.6%-100%). There were relatively low levels of IGT counseling during PCP visits (53.8%-54.5%). In the BRIDGE+ population, a larger proportion of patients scheduled an iPPV than a PCP visit (59.5% vs 29.1%), but attendance rates at scheduled iPPV visits were much lower than those for PCP visits (48.9% vs 100%).

## Discussion

### Principal Results

Implementing BRIDGE, an SMS text messaging support program with educational and behavioral prompts focusing on postpartum transitions for patients with IGT during pregnancy, resulted in increased completion of an HbA_1c_ test within 1 year postpartum and increased scheduling of a PCP visit by 12 weeks postpartum. The addition of an iPPV to the SMS text messaging support improved the completion of HbA_1c_ testing by 6 weeks postpartum, but this effect did not persist at 1 year postpartum and did not impact PCP visit scheduling.

### Comparison With Prior Work

To our knowledge, this is the first study examining the impact of an intervention on postpartum transition milestones for patients diagnosed with IGT in pregnancy, who often do not receive tailored support during pregnancy and the immediate postpartum period through specialized programming given to patients with HDP or GDM (eg, transition clinics, remote blood pressure monitoring programs, and diabetes in pregnancy programs). This is an important population to target as these patients experience higher rates of negative interpregnancy and long-term health outcomes [28-30].

While there is a paucity of literature focusing on patients with IGT, postpartum transitions for patients with GDM have been well studied. Postpartum glucose screening rates within 12 weeks postpartum for patients with GDM (primarily assessed via completion of a 2-hour glucose tolerance test rather than HbA_1c_ completion by 6 weeks postpartum, based on guidelines) range from 7% to 59% in the literature, which are similar to the 6-week HbA_1c_ completion rate in historical and BRIDGE populations [31-34]. The PCP visit attendance rates for both the historical population and BRIDGE population were similar to published rates, which range from 22% to 60% [15,35]. Multiple systematic reviews assessing the impact of reminder interventions (eg, emails, phone calls, and mail) on postpartum glucose screening rates for patients with GDM have shown potential benefits [36-38]. A randomized controlled trial of a text message intervention targeting primary care transitions for patients with GDM and HDP improved PCP visit completion by 18.7%, which is similar to the difference in PCP visit attendance between the historical and BRIDGE populations [15]. Overall, this consistency with existing literature provides reassurance that the BRIDGE intervention may be generalizable to other settings.

This program evaluation demonstrated that the integration of iPPV increased HbA_1c_ completion in the first 12 weeks postpartum, but this improvement did not persist until 1 year postpartum and did not impact PCP visit scheduling. Patient engagement may play a role in this transient impact of iPPV integration on desired postpartum milestones. While the iPPV scheduling rate was nearly double the PCP visit scheduling rate, the number of individuals who attended the iPPV was similar to the number of individuals who attended the PCP visit. Although the authors were insufficiently powered to assess the relationship between iPPV and PCP visit attendance, it is also possible that patients who completed an iPPV perceived a decrease in the usefulness of PCP visits in the first year postpartum because they had already received IGT-counseling and screening via HbA_1c_ completion from a prenatal care provider.

This program evaluation highlights areas for improvement in the quality of the postpartum transition for patients with complications of pregnancy, as a large proportion of PCP visits in both the historical and BRIDGE populations did not include counseling regarding IGT. Per existing literature, potential contributing factors to this gap in primary care transitions may be related to communication breakdowns between the obstetric and primary care teams regarding pregnancy complications that may influence long-term counseling or management, awareness and knowledge of GDM guidelines among PCPs, and patients’ recall of providers’ counseling regarding long-term risks, among other factors [39,40]. Potential solutions include enhanced support for care coordination (eg, expedited referral pathways to primary care and patient navigation services), improved handoffs between obstetric and PCP teams (eg, standardized documentation of pregnancy complications), and reminder systems [41].

Our efforts to effectively implement BRIDGE highlighted many deep-rooted challenges with primary care transitions for pregnant patients. From a health care system standpoint, PCP access was limited by both health insurance specifications and reduced capacity in the primary care workforce. This limited the ability to successfully schedule PCP visits for postpartum patients without targeted efforts in this area. In addition, patients reported negative social drivers of health (eg, lack of childcare support, transportation support, or challenges with newborn care such as a city-wide formula shortage) as well as physical or mental health challenges that served as barriers or competing priorities for patients attempting to complete postpartum transition milestones. These challenges have been well characterized in both quantitative and qualitative analyses within the literature and highlight the need for a multilevel approach to optimizing postpartum transitions of care, addressing individual, health care system, and societal barriers [42-44].

### Limitations and Strengths

The study had several key limitations. As a nonrandomized pre-post implementation study, our analysis cannot account for unmeasured confounders. The BRIDGE population had higher-risk metabolic comorbidities than the historical population (eg, history of IGT in prior pregnancy), but the overall cohort size was much smaller. As only 8 participants declined enrollment in BRIDGE, the study does not suspect that this difference in cohort size is reflective of selection bias but may be due to other unmeasured factors contributing to decreased rates of IGT in the contemporary population. The COVID-19 pandemic also introduced constraints to the preimplementation or postimplementation design that increase the risk of temporal bias. For example, the historical population in this study was not subject to the systemic changes in health care post–COVID-19, such as the rising use of telemedicine [45], which may have improved access to care [46]. Finally, the study was unable to recruit the target number of participants despite extending the planned enrollment period due to decreases in the prevalence of IGT in the population. The smaller sample size raises some concern regarding the precision and robustness of estimates in the primary analysis, as well as with the multiple imputation analysis. A larger, randomized trial will be required to ascertain the definitive impact of this intervention, though this study provides positive preliminary results.

There are also some scalability concerns with the BRIDGE program. Specifically, the iteration of the BRIDGE program used in this study was resource intensive, involving the manual enrollment of participants by study staff using EMR review; however, if proven effective, these resource intensive steps could be automated, as has been done with other W2H programs in our institution [47]. In addition, other institutions may not have access to the staffing capacity required to support other aspects of the program, such as social work support or IGT-focused postpartum visits. Finally, the study enrolled only English-speaking patients at a single tertiary care institution in the northeastern United States, which limits generalizability.

The study may also be prone to bias due to missing data. For patients with out-of-network PCPs, the study had extremely limited data on PCP visit scheduling and attendance. Phone interviews were attempted for BRIDGE patients to obtain self-reported data but had a low response rate (n=10). Phone interviews were deferred for the historical population because of concerns about recall bias. It is possible, therefore, that the PCP visit scheduling and attendance rates among patients with out-of-network PCPs in the historical population were under-reported. This would bias the results toward programmatic impact. However, reassuringly, the beneficial impact of BRIDGE on PCP visit scheduling persists when looking at patients with in-network PCPs alone, where complete access to primary care data is present.

The study also had many strengths. First, the development of the BRIDGE program used robust input from interdisciplinary teams spanning behavioral health, digital health, and communications expertise [48]. In addition, the preimplementation and ongoing implementation work with prenatal care sites allowed the authors to optimize the effectiveness of the program using flexible and varied implementation strategies [49], based on an expansive understanding of the barriers faced by patients and providers attempting to achieve a successful postpartum transition. Finally, the population was racially and socioeconomically diverse with varied experiences with PCP care entering pregnancy, which may make their experiences more generalizable to other populations.

Future directions for research could include additional assessments of implementation outcomes (eg, fidelity and acceptability) to ascertain contributing factors to the difference in outcomes between BRIDGE− and BRIDGE+ [50]. Following this work, a formal implementation trial of BRIDGE where groups are randomized to the intervention, which would account for any unmeasured confounding in this study [51]. Planning for that trial should include phone or in-person surveys or other strategies to obtain complete information on primary care transitions for individuals with out-of-network PCPs. Consideration should also be given to adapting BRIDGE to be applicable to more diverse or resource-limited settings [52]; potential strategies could include the automation of participant enrollment, integration of iPPV content into routine postpartum visits (thus eliminating the need for additional staffing), the conversion of iPPV from an in-person to telehealth visit (thus eliminating the need for additional clinic capacity), and the cocreation of adapted messaging in other languages alongside community partners.

### Conclusions

In this nonrandomized pre-post implementation study, SMS text messaging support improved postpartum transitions of care for patients diagnosed with IGT in pregnancy, though the quality of the transitions to primary care remained low with poor rates of IGT counseling by PCPs. SMS text messaging support with educational and behavioral prompts tripled completion rates of HbA_1c_ screening within 1 year postpartum and doubled the scheduling rate for PCP visits by 12 weeks postpartum. While attendance at scheduled PCP visits was very high, less than 60% of PCP visits included IGT counseling, highlighting key areas for improvement in the quality of postpartum transitions to primary care. This study also highlighted a complex array of individual, systemic, and societal barriers to achieving desired postpartum milestones. A larger randomized trial is needed to definitively ascertain the impact of this SMS text messaging intervention. Optimizing postpartum transitions of care for populations with longitudinal metabolic risk after pregnancy will require a multilevel approach, of which text message support could be an important component.

## Supplementary material

10.2196/76493 Multimedia Appendix 1 Multiple imputation analysis for primary care visit scheduling and attendance.

## Abbreviations

aOR: adjusted odds ratio
BRIDGE: Better Follow-up of Impaired Glucose Tolerance
CONSORT: Consolidated Standards of Reporting Trials
EMR: electronic medical record
GDM: gestational diabetes mellitus
HbA_1c_: hemoglobin A_1c_
HDP: hypertensive disorders of pregnancy
IGT: impaired glucose tolerance
iPPV: IGT-focused postpartum visit
PCP: primary care provider
W2H: Way2Health
