# Supplementary material for: Text Messaging Support for Patients Diagnosed With Impaired Glucose Tolerance During Pregnancy: Nonrandomized Pre-Post Implementation Study Assessing Impact on Postpartum Transitions of Care
Source: J Med Internet Res. 2026 Jun 9;28:e76493. doi: 10.2196/76493 (PMC13249060; doi:10.2196/76493)
Supplement: Multimedia Appendix 1 [file jmir-v28-e76493-s001.docx]

APPENDIX

Table 5: Multiple imputation analysis for primary care visit scheduling and attendance

| **Postpartum Transitions** | **Historical population**  **(n=342)** | **BRIDGE population**  **(n=161)** | **aOR (95% CI)^e^** | **Multiple imputation** |
| --- | --- | --- | --- | --- |
| PCP visit scheduling, n(%), *95% CI*  In network PCPs | 41 (12.0%)  *9.0%-15.9%* | 44 (27.3%)  *21.0%-34.7%* | 8.30 (3.77-18.2) |  |
| All patients^d^ | 41 (12.0%)  *9.0%-15.9%* | 50 (31.0%)  *24.4%-38.6%* | 9.58 (4.39-20.9) | 9.49 (4.60-19.6) |
| PCP visit attendance, n(%), *95% CI*  In network PCPs    All patients | 38 (92.7%)  *80.6%-97.5%*  38 (92.7%)  *80.6%-97.5%* | 42 (95.4%)  *84.9%-98.7%*  48 (96%)  *86.5%-98.9%* | 1.81 (0.28-11.7)  2.11 (0.33-13.6) | 2.05 (0.24-17.4) |

| **Postpartum Transitions** | **BRIDGE- (n=82)** | **BRIDGE+**  **(n=79)** | **aOR (95% CI)** | **Multiple imputation** |
| --- | --- | --- | --- | --- |
| PCP visit scheduling, n(%), *95% CI*  In network PCPs | 21 (25.6%)  *17.4%-36.0%* | 23 (29.1%)  *20.3%-39.9%* | 0.47 (0.11-2.09) |  |
| All patients^c^ | 24 (29.3%)  *20.5%-39.9%* | 26 (32.9%)  *23.6%-43.9%* | 0.46 (0.11-2.00) | 0.49 (0.10-2.25) |
| PCP visit attendance, n(%), *95% CI*  In network PCPs      All patients | 19 (90.4%)  *71.1%-97.3%*  22 (91.6%)  *74.2%-97.7%* | 23 (100%)  *85%-100%*  26 (100%)  *87%-100%* | ---  --- | ---  --- |
